# Can We Predict Oral Antibiotic Treatment Failure in Children with Fast-Breathing Pneumonia Managed at the Community Level? A Prospective Cohort Study in Malawi

**DOI:** 10.1371/journal.pone.0136839

**Published:** 2015-08-27

**Authors:** Carina King, Eric D. McCollum, Limangeni Mankhambo, Tim Colbourn, James Beard, Debbie C. Hay Burgess, Anthony Costello, Raza Izadnegahdar, Norman Lufesi, Gibson Masache, Charles Mwansambo, Bejoy Nambiar, Eric Johnson, Robert Platt, David Mukanga

**Affiliations:** 1 Institute for Global Health, University College London, London, United Kingdom; 2 Department of Pediatrics, Division of Pulmonology, Johns Hopkins School of Medicine, Baltimore, Maryland, United States of America; 3 Parent and Child Health Initiative, Lilongwe, Malawi; 4 Bill & Melinda Gates Foundation, Seattle, Washington, United States of America; 5 Acute Respiratory Infection Unit, Ministry of Health, Lilongwe, Malawi; 6 Ministry of Health, Lilongwe, Malawi; 7 Center for Health Research, Kaiser Permanente Northwest, Portland, Oregon, United States of America; 8 McGill University, Montreal, Canada; University of Barcelona, SPAIN

## Abstract

**Background:**

Pneumonia is the leading cause of infectious death amongst children globally, with the highest burden in Africa. Early identification of children at risk of treatment failure in the community and prompt referral could lower mortality. A number of clinical markers have been independently associated with oral antibiotic failure in childhood pneumonia. This study aimed to develop a prognostic model for fast-breathing pneumonia treatment failure in sub-Saharan Africa.

**Method:**

We prospectively followed a cohort of children (2–59 months), diagnosed by community health workers with fast-breathing pneumonia using World Health Organisation (WHO) integrated community case management guidelines. Cases were followed at days 5 and 14 by study data collectors, who assessed a range of pre-determined clinical features for treatment outcome. We built the prognostic model using eight pre-defined parameters, using multivariable logistic regression, validated through bootstrapping.

**Results:**

We assessed 1,542 cases of which 769 were included (32% ineligible; 19% defaulted). The treatment failure rate was 15% at day 5 and relapse was 4% at day 14. Concurrent malaria diagnosis (OR: 1.62; 95% CI: 1.06, 2.47) and moderate malnutrition (OR: 1.88; 95% CI: 1.09, 3.26) were associated with treatment failure. The model demonstrated poor calibration and discrimination (c-statistic: 0.56).

**Conclusion:**

This study suggests that it may be difficult to create a pragmatic community-level prognostic child pneumonia tool based solely on clinical markers and pulse oximetry in an HIV and malaria endemic setting. Further work is needed to identify more accurate and reliable referral algorithms that remain feasible for use by community health workers.

## Background

Globally, pneumonia is the leading infectious cause of death amongst children under five, with an estimated 0.9 million deaths annually, and the highest burden found in Africa [1, 2]. While there have been reductions in pneumonia mortality over the last decade and several cost-effective interventions are available, such as *Haemophilus influenzae B* (HiB) and pneumococcal conjugate vaccines (PCV) [3, 4], effective care seeking and case management will still be needed to significantly lower mortality.

The World Health Organisation (WHO) integrated community case management (iCCM) guidelines clinically stratify pneumonia into either non-severe pneumonia (fast breathing only) or severe pneumonia requiring referral (presence of chest indrawing or danger signs) [5]. Non-severe cases—or ‘fast-breathing pneumonia’—are treated at home with oral antibiotics. Implementation of standardized treatment and referral guidelines has been associated with a 36% reduction in pneumonia mortality [6]. A potential strategy to further reduce mortality among children with fast-breathing pneumonia would be to identify those at risk of oral antibiotic treatment failure and higher mortality rates [7], for early referral when they are potentially more responsive to interventions.

Treatment failure is the persistence of symptoms or deterioration following antibiotic initiation [8]. Common causes of treatment failure include incorrect initial diagnosis, host comorbidities such as HIV infection and malnutrition, and viral or antibiotic-resistant causative organisms [9]. Several predictive tools have been developed to distinguish those children with pneumonia who are at higher risk of adverse outcomes (i.e. treatment failure and death) [10, 11]. Factors commonly found to be associated with treatment failure are young age, fast breathing, hypoxemia (or a peripheral oxygen saturation (SpO_2_) <90%) and poor antibiotic adherence [12–16]. To date these studies have been predominantly focused at the hospital level, among severe and very-severe pneumonia patients, the outcome of death, and used data collected by facility-based healthcare workers. A recent literature review for risks of oral antibiotic treatment failure found a considerable evidence gap in understanding the predictors of treatment failure at the community level and with fast-breathing pneumonia, specifically from the African region [17].

A referral algorithm that could be implemented at the community-level (e.g. as an mHealth tool) presents an opportunity for further reduction in pneumonia morbidity and mortality, through more rapid referral of higher risk children when they are more amenable to alternative treatments. The aim of this study was to develop a pragmatic prognostic algorithm for treatment failure in children diagnosed with fast-breathing pneumonia by community health workers (CHWs), treated with oral co-trimoxazole (the first line treatment during the study period) in the community.

## Materials and Methods

### Setting

We conducted a prospective cohort study in Malawi from September 2013 to June 2014, nested within a larger parent study assessing the impact of PCV introduction in Malawi. This study was conducted in sub-populations of two districts in the central region of Malawi (Mchinji and Lilongwe). The populations covered were all rural, with mobile phone ownership of 35% and the populations were predominantly subsistence farmers [18].

The primary health care system in rural Malawi consists of government employed CHWs, called Health Surveillance Assistants locally. CHWs receive 12 weeks basic training delivered by the government (including iCCM), and they run designated village clinics one or two times per week as well as conducting house visits and outreach campaigns. These CHWs cover catchment areas of approximately 1,200 people, although this varies according to region, and are supervised by senior CHWs based at health facilities.

### Data Collection

Patients were recruited at weekly village clinics run by 34 CHWs, 18 in Mchinji and 16 in Lilongwe rural, with a catchment area of approximately 50,000 people and 8,500 children under five. CHWs were responsible for the initial clinical assessment, following slightly modified iCCM guidelines with concurrent diagnoses and treatments allowed; in Malawi HIV and malaria rapid diagnostic testing (RDTs) are not fully implemented at the community-level and it is common for acute fevers to be presumptively treated as malaria [19]. This cohort of CHWs had not been trained to conduct paediatric HIV or rapid malaria testing.

If diagnosed with pneumonia, information from the clinical assessment (including pulse oximetry with Lifebox®) was recorded on case report forms (CRFs) by the CHW, along with their referral or treatment decision. To measure PCV effectiveness, the parent PCV study required a consistent clinical pneumonia definition applied across the community, health centre, and hospital health system levels. Therefore, the CHW CRFs included additional respiratory signs associated with more severe disease and routinely collected at the facility level, but not previously included at the community level, including nasal flaring, head nodding, and grunting. Pulse oximetry and the CRFs were introduced among these CHWs in late 2011 as part of the parent PCV study and were also used for this sub-study, both are not currently standard care at this level in Malawi [20]–all other aspects of care were routine. CHWs had received training on the CRFs, pulse oximetry and clinical assessments in 2011, and attended monthly mentorship meetings and annual refresher trainings with local senior clinical staff in the two years preceding and throughout this study, to ensure quality data collection. CRFs were pre-printed with a unique barcode and were submitted monthly to the CHW supervisors who checked and submitted them to research project staff. This data was entered into a Microsoft Access database, with regular data cleaning and checking.

Although CHWs are recommended to follow up children after pneumonia treatment, this is not done routinely in actual practice, and this study required routine, timely, and rigorous outcome assessments. Community-level outcome assessments were not included in the parent PCV study. For each village clinic, we therefore employed local village level data collectors (VDCs) to recruit and conduct routine, timely, and comprehensive follow up interviews. VDCs received training on conducting a clinical assessment for pneumonia signs and symptoms, using a pulse oximeter, the study protocol and questionnaires. Clinical assessment training included the use of videos, demonstrations, practical sessions and supervised assessments with real cases at local health centres. All CHWs also attended the training. A one month field pilot (August 2013) was conducted, followed by a half day refresher training for all VDCs and CHWs prior to study recruitment.

Patient recruitment was done by the VDCs at the village clinics (on ‘Day 0’). Patients were eligible for recruitment if the CHW had diagnosed them with fast-breathing pneumonia and prescribed oral antibiotics for home treatment. VDCs followed up patients at their homes on day 5 and day 14. If the patient was not located on day 5, the VDCs re-visited the home on day 7. At the follow up interviews, VDCs conducted a clinical assessment of the child (including pulse oximetry), asked about additional care seeking and antibiotic adherence. We used smart phones for follow up data collection, with data downloaded onto field supervisor’s laptops every 2 weeks and cleaning checks run each month. Data was linked with the CHW filled CRFs through the unique barcode, which the VDCs scanned to register a case at the village clinic.

Several *data quality assurance* exercises were carried out to ensure reliable and consistent data, including: 1) an initial vital signs standardization audit conducted by a paediatric pulmonologist, which all VDCs passed. 2) Random GPS spot checks by field supervisors of follow up locations. 3) All VDCs had at least one interview supervised by a pediatrician to ensure standardized clinical assessments including oximetry, in which all performed satisfactorily. 4) Monthly data review and feedback meetings, to highlight and address data quality issues. 5) Regular shadowing of interviews by field supervisors, with concurrent recording of clinical signs. 6) Remote clinical validation for the presence of lower chest indrawing from a random sample of VDC collected video clips.

### Definitions

#### Cohort

all children aged 2–59 months with CHW-diagnosed WHO fast-breathing pneumonia who were adherent to home oral co-trimoxazole treatment. First line treatment for fast-breathing pneumonia in Malawi is a 5-day BD (twice a day) course of co-trimoxazole (where a dose is: 1/2 480mg tablet for 2–11 months; 1 480mg tablet for 12–59 months).

#### Fast-breathing pneumonia

defined according to the 2013 iCCM guidelines[5], as the presence of cough or difficult breathing and fast breathing (>50 breaths per minute for infants 2–11 months; >40 breaths per minute for children 12–59 months), in the absence of any danger signs or lower chest indrawing. Danger signs were recorded based on caregiver report and clinical observation by the CHWs at presentation, and included: vomiting everything, unable to feed, convulsions in the previous 24 hours, sleepy or unconscious, SpO_2_ <90%, and grunting, nasal flaring and head nodding as signs of severe respiratory distress. All children who were severely malnourished (mid-upper arm circumference (MUAC) <11.5cm) were referred.

#### Treatment failure

defined as the presence of any of the following on day 5 of follow up (the day after the final dose of antibiotics): fast breathing for age, axillary temperature >37.5°C, lower chest indrawing, any danger sign (as defined above), change of antibiotic, hospital admission or death (adapted from Fox et al. [21]). Change in antibiotic was based on caregiver report, with a named antibiotic prescribed by any level of clinical staff, and where possible visually verified by the VDC. VDCs had received training on common antibiotics and their trade names, and this information was printed for them to take to interviews for reference.

#### Adherence

defined as completing at least 80% of the prescribed antibiotics and confirmed by visual examination of remaining antibiotics by the VDC. We restricted the model to ‘adherent’ children only, as non-adherence has been shown to be associated with treatment failure in previous studies; the aim of this study was to make a referral tool based on clinical presentation only.

### Analysis

We described the cohort, including levels of missing data. The following variables were determined *a priori* for inclusion in the prognostic model based on a literature and expert panel review and practicality for use at the community level: oxygen saturation; respiratory rate; fever (axillary temperature >38°C); malnutrition, as determined by MUAC measurements; age; PCV-13 and pentavalent vaccination (including HiB). We also decided to include concurrent clinical malaria diagnosis. The continuous variables were categorized before inclusion into the model, based on substantive knowledge as follows: oxygen saturation of SpO_2_ 90–94% and ≥95%; MUAC as moderate malnutrition (11.5–13.5cm) and normal nutrition (≥13.5cm); age as 2–5, 6–11, and 12–59 months; respiratory rate as fast (>50 breaths per minute for 2–11 months, >40 breath per minute for 12–59 months) and very fast breathing (>70 breaths per minute for 2–11 months, >60 breath per minute for 12–59 months).

Multivariable logistic regression was used to create the prognostic model, retaining all pre-defined risk variables. Missing data were imputed using multiple imputation with chained equations [22], assuming that data was missing at random. The model equation was validated through bootstrapping. Model performance was evaluated using: pseudo R^2^; the Hosmer-Lemeshow goodness of fit test (p-value>0.05 indicates good fit); c-statistic (scale of 0.5–1.0, with 1.0 being perfect discrimination); slope shrinkage in the internal validation (scale of 0.0–1.0, with 1.0 indicating no over-fitting). Additional information is found in S1 Text. The patient’s log odds of treatment failure as derived from the model equation were converted into the probability that they failed treatment, using the following equation:

Probability of treatment failure= exp(betaX)/(1+exp(betaX))

#### Sample size

The sample size calculation was based on the requirement of 10 treatment failure cases per degree of freedom in the model, therefore with the eight candidate predictors evaluated using 11 degrees of freedom, we required a sample of at least 110 treatment failures.

#### Sensitivity analyses

We performed sensitivity analyses to compare: 1) the baseline characteristics between those lost to follow up and those included in the final analysis; 2) check for outlying CHWs to determine whether data collection inaccuracies could give rise to spurious prediction results. For baseline clinical observations we calculated the mean, standard deviation, minimum, 25^th^ percentile, median, 75^th^ percentile and maximum values by CHW. CHWs were flagged if for any variable, the 25^th^ and 75^th^ percentiles were equal and the model was re-fit without the data from these CHWs. All descriptive analysis was done using Stata SE11 and statistical analysis was done using R version 3.1.1 [23].

### Ethics

Informed consent for study recruitment and prospective follow up was sought from the child’s accompanying care-giver at the initial clinical assessment and was confirmed at each subsequent follow up with the care-giver present. The study information sheet was read aloud by the VDC before starting the interview. Consent was given verbally and recorded in the electronic form on the smart phone. Verbal consent was sought as literacy is low in this population. Ethical approval for this informed consent procedure and the overall study was granted by the National Health Sciences Research Committee in Malawi (reference: 941).

## Results

**Fig 1:**
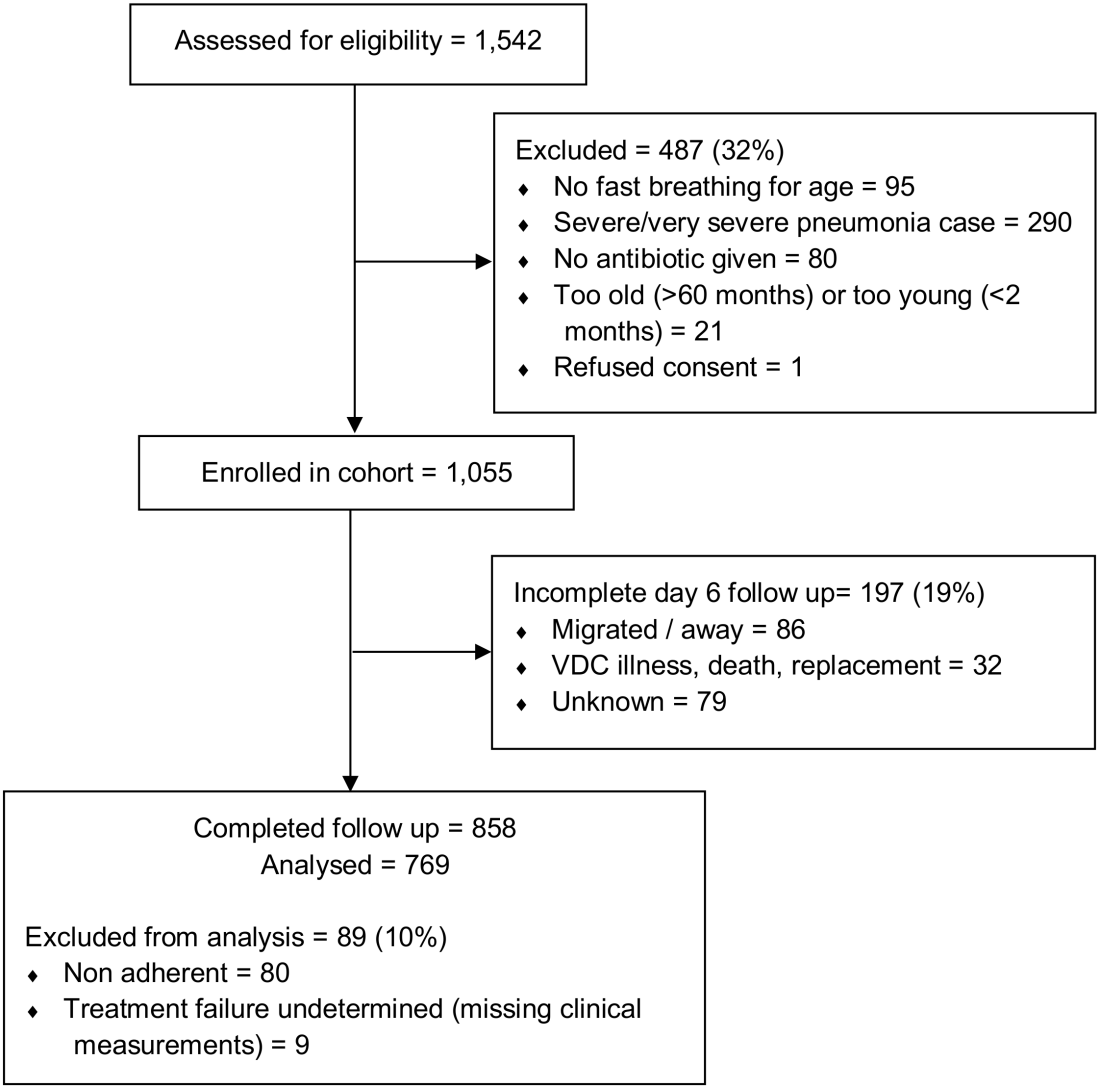
Participant flow diagram for inclusion in the prognostic algorithm model.

The VDCs assessed 1,542 children diagnosed with fast-breathing pneumonia by the CHWs for enrollment over a 9 month period, of which 1,055 were eligible. Reasons for ineligibility were: presence of lower chest indrawing and/or danger signs (60%), no fast breathing (20%), children were not given antibiotics (16%) or children were too old or too young (4%). A total of 197 children (19%) were lost to follow-up; Table 1 shows their baseline characteristics separately from those with follow-up data. Among the 858 children with complete follow-up, we included 769 in the final analysis (Fig 1). Of these, 114 had treatment failure at day five of follow-up, a risk of 14.8% (95% CI: 12.3 to 17.3). Of these treatment failure cases, 29 (25.4%) had not recovered at day 14 and of those without treatment failure we observed an additional 25 children with relapse at day 14 of follow up (3.8%; 95% CI: 2.8 to 6.2). The main reasons for being classified as a treatment failure case were: persistence of fast breathing (65%); grunting (12%); fever (9%) and prescription of an alternative antibiotic (9%)—Table 2.

**Table 1:** Baseline patient characteristics, including predictors of treatment failure for children enrolled in the cohort by loss to follow-up (sensitivity analysis 1)

**Characteristic**	**Complete follow-up (n = 769)**	**Lost to follow-up (n = 197)**
	**Mean (SD)**	**Mean (SD)**
	**Proportion (% missing)**	**Proportion (% missing)**
Age (months)	21.7 (14.7)	22.8 (14.9)
12 to 59 months	70.3% (0.9%)	75.1% (2.0%)
6 to 11 month	16.5%	13.0%
2 to 5 months	13.1%	11.9%
Respiratory rate: 2–11 months (breaths/min)	56.2 (5.1)	57.1 (5.9)
12–59 months (breaths/min)	47.5 (7.1)	47.7 (5.8)
Very fast	1.1% (6.1%)	4.0% (7.1%)
Oxygen saturation (%)	96.4 (1.9)	96.7 (2.0)
Abnormal (90–94%)	15.3% (1.4%)	12.8% (0.5%)
Temperature (°C)	37.1 (1.0)	37.2 (1.0)
Fever ≥38°C	18.2% (4.2%)	19.8% (5.0%)
MUAC (cm) *	14.7 (1.4)	15.0 (1.4)
Moderately malnourished (<13.5 cm)	17.1% (19.5%)	10.1% (19.8%)
	**Proportion (% missing)**	**Proportion (% missing)**
District: Mchinji	45.5% (0%)	39.1% (0%)
Sex: Female	52.7% (2.0%)	52.6% (0.5%)
Pentavalent vaccine: 3 doses	92.5% (6.4%)	90.3% (6.1%)
PCV13 vaccination: 3 doses *	78.6% (23.0%)	65.9% (36.0%)
†Concurrent malaria diagnosis	40.2% (0.3%)	-

The two study areas were in Mchinji and Lilongwe rural, both in the central region of Malawi. SD: standard deviation; MUAC: mid-upper arm circumference; PCV13: 13-valent pneumococcal conjugate vaccine.

*p-value<0.05 using chi2 test or t-test.

^†^Concurrent diagnosis was confirmed at follow-up so those lost to follow-up do not have this information. Malaria diagnosis was clinical and made at the same time as the pneumonia diagnosis by the CHWs.

**Table 2:** Distribution of World Health Organization criteria for diagnosing treatment failure at day 5 of follow up

Treatment failure criteria	Number (%)
	(n = 114/769)
Treatment failure	114 (14.8%)
Fast Breathing†	74 (64.9%)
SpO_2_ <90%	7 (6.1%)
Axillary temperature >37.5°C	10 (8.8%)
Lower chest indrawing	6 (5.3%)
Danger signs:	26 (22.8%)
Vomiting everything	4 (3.5%)
Nasal Flaring	5 (4.4%)
Grunting	14 (12.3%)
Head nodding	0
Convulsions	2 (1.8%)
Sleepy/unconscious	1 (0.9%)
Unable to feed	2 (1.8%)
Admitted to hospital	2 (1.8%)
Prescribed alternative antibiotic	10 (8.8%)

‘Admitted to hospital’ and ‘prescribed alternative antibiotic’ were based on care giver report. SpO_2_: peripheral oxygen saturation

Children fulfilling more than one criteria will appear more than once

^†^Fast breathing defined as >50 breaths per minute in infants aged 2–11 months, and >40 breaths per minute in children aged 12–59 months.

Odds ratios, following imputation for missing values, for the eight pre-defined predictors of treatment failure are reported in Table 3. Concurrent clinical malaria diagnosis (aOR: 1.62; 95% CI: 1.06, 2.47) and moderate malnutrition (aOR: 1.88; 95% CI: 1.09, 3.26) were both significantly associated with treatment failure. The prediction model’s eight characteristics explained 5.1% of the variation in the risk of treatment failure (R^2^ value). The model equation is:

$$\begin{array}{l} Log\;odds\;of\;treatment\;failure\;\\ =\;-2.475\;+\;\left(age\;6-11\;months\;x\;-0.315\right)\;+\;\left(age\;2-5\;months\;x\;0.057\right)\;\\ +\;\left(very\;fast\;breathing\;x\;0.001\right)\;+\;\left(hypoxemia\;x\;0.441\right)\;+\;\left(fever\;x\;-0.495\right)\;\\ +\;\left(moderately\;malnourished\;x\;0.633\right)\;+\;(1-2\;doses\;pentavalent\;vaccine\;x\;\\ -1.267)\;+\;\left(3\;doses\;pentavalent\;vaccine\;x\;0.189\right)\;+\;\left(1-2\;doses\;PCV\;x\;0.621\right)\;\\ +\;\left(3\;doses\;PCV\;x\;0.284\right)\;+\;\left(concurrent\;malaria\;diagnosis\;x\;0.480\right) \end{array}$$

**Table 3:** Predictors of treatment failure after multiple imputation: odds ratios and confidence intervals.

Predictor	Odds ratio (95% CI)
Age (grouped linear)	
12 to 59 months	1.0
6 to 11 months	0.73 (0.39, 1.35)
2 to 5 months	1.06 (0.53, 2.12)
Respiratory rate (indicator)	
Fast	1.0
Very fast	1.00 (0.48, 2.08)
Oxygen saturation (grouped linear)	
Normal (≥95%)	1.0
Abnormal (90–94%)	1.55 (0.90, 2.67)
Fever (indicator)	
No	1.0
Yes (≥38°C)	0.61 (0.34, 1.11)
MUAC (indicator)HYPERLINK *	
Well nourished (≥13.5cm)	1.0
Moderately malnourished (<13.5cm)	1.88 (1.09, 3.26)
Pentavalent vaccination	
No doses	1.0
1–2 doses	0.28 (0.02, 4.07)
3 doses	1.21 (0.34, 4.29)
PCV13 vaccination	
No doses	1.0
1–2 doses	1.86 (0.26, 13.06)
3 doses	1.33 (0.62, 2.87)
Concurrent malaria diagnosis*	
No	1.0
Yes	1.62 (1.06, 2.47)

CI: confidence interval; MUAC: mid-upper arm circumference; PCV13: 13 valent pneumococcal conjugate vaccine. ‘Fast’ respiratory rate was defined as >50 breaths per minute for infants aged 2–11 months and >40 breath per minute for 12-59months, and ‘very fast’ was >70 breaths per minute for infants aged 2–11 months and >60 breath per minute for 12-59months.

*p-value<0.05

**Fig 2:**
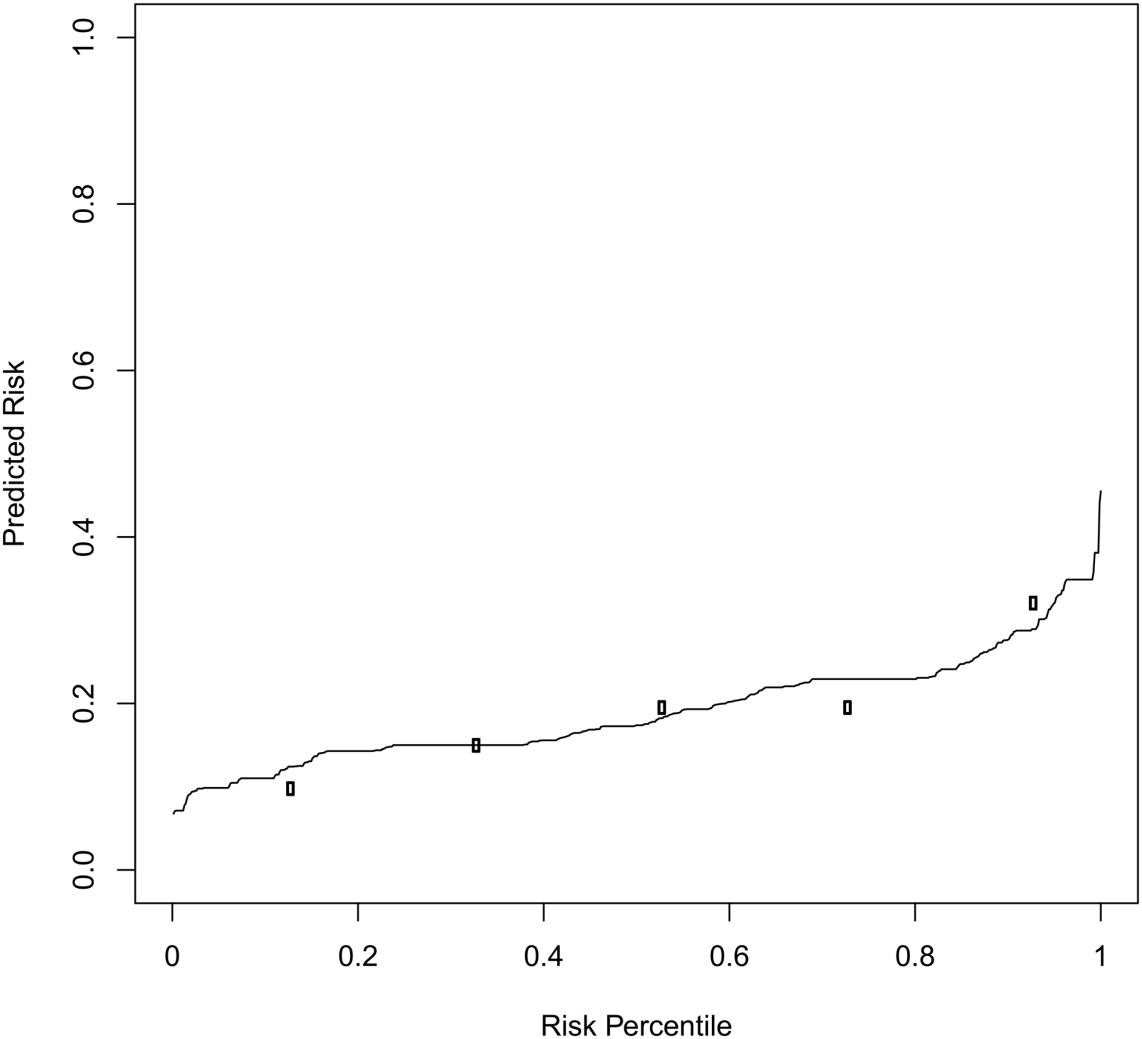
Risk predictiveness curve. The solid line indicates children’s predicted risk of treatment failure. For each quintile of predicted risk, we plotted the observed risks of treatment failure (open circles). The agreement between the predicted and observed risks measures the calibration.

The predicted risk of treatment failure was plotted with a risk predictiveness curve (Fig 2). The predicted risk of treatment failure increased gradually until the 80^th^ percentile. The observed risks of treatment failure for each quintile of predicted risk were plotted at the mid-point of the quintile. Children in the highest-risk quartile were 3.19 times more likely to fail oral co-trimoxazole than children in the lowest-risk quartile, a measure of discrimination. The c-statistic (0.56 after bootstrap validation) demonstrated that the model had a low ability to detect subtle differences or discriminate. We found evidence of adequate calibration as the observed risks agreed approximately with the predicted risks for each quintile. However, the Hosmer-Lemeshow goodness of fit test showed some evidence of lack of fit of the model (P = 0.04).

**Fig 3:**
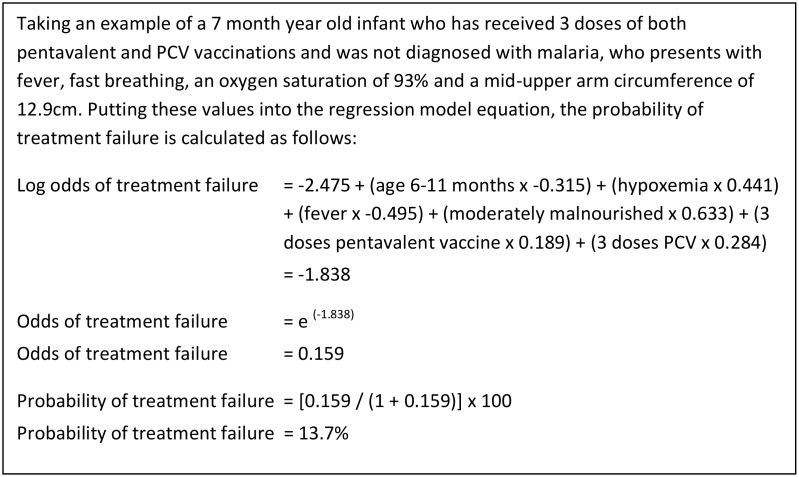
Example of calculating probability of treatment failure.

If CHWs referred those children in the top risk quintile (i.e. any child who’s score indicated a predicted risk ≥80^th^ percentile), 33.3% (95% CI: 24.4, 41.6) of the treatment failure cases would be referred; this is the sensitivity. The same decision rule would result in a specificity of 82.4% (95% CI: 79.5, 85.3), a positive predictive value of 24.8% (95% CI: 18.0, 31.6) and negative predictive value of 87.7% (95% CI: 85.1, 90.3). While the sensitivity and specificity are properties of the prediction model and the decision rule for referral, the predictive values depend on the incidence of treatment failure in a given population and setting. Fig 3 gives a worked example.

**Fig 4:**
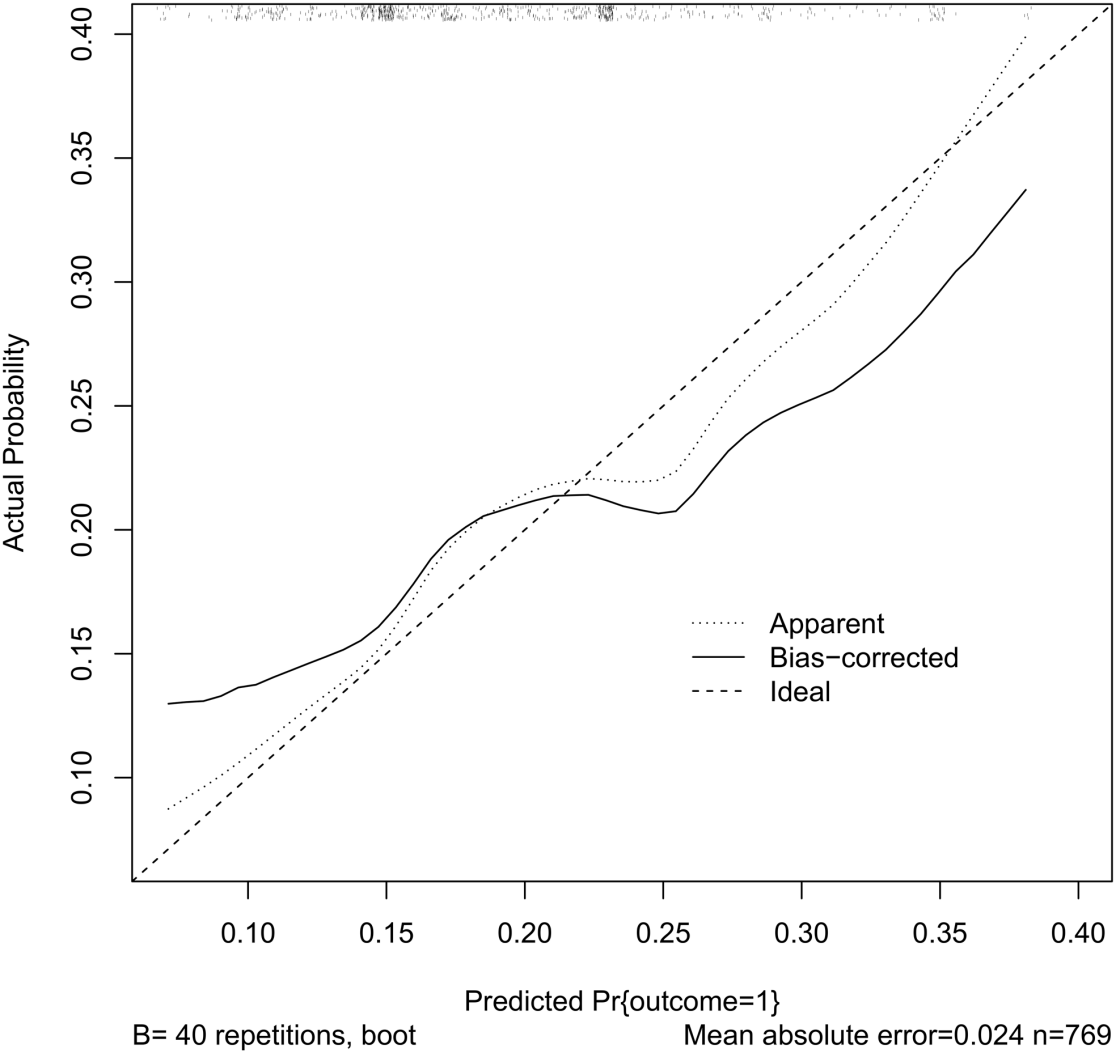
Observed and predicted treatment failure. The dotted line plots the observed vs. predicted probability of treatment failure the solid line is a bias-corrected version (40 bootstrap repetitions). The rug plot along the top of the graph indicates the distribution of the predicted probabilities.

Based on the internal (statistical) validation, we found some evidence of over-fitting the logistic model to the data because the slope-shrinkage statistic was 0.6. Fig 4 demonstrates the discrepancy between the apparent and bootstrap-corrected curves. For the sensitivity analysis of outlying CHW measurements, we found no CHWs with suspicious data.

## Discussion

We found a treatment failure rate of 14.8% at day 5 of follow up, for fast-breathing pneumonia treated with co-trimoxazole. This is consistent with rates found in previous studies of fast-breathing pneumonia treatment failure with co-trimoxazole, with rates ranging from 11–21%, albeit from studies conducted in South Asia at the facility level, and not with CHWs [15, 24]. The model performed relatively poorly with a c-statistic of 0.56 and R^2^ of 5.1%. We found the best predictors for failure were a concurrent clinical malaria diagnosis and moderate malnutrition, while predictors such as young age and respiratory rate did not show any significant associations. Possible explanations of our findings include: lack of key variables (e.g. HIV status and laboratory-confirmed malaria); challenges in community diagnosis of pneumonia; and quality of antibiotics, adherence and additional care seeking.

In the context of this stud y setting, we were unable to reliably or ethically collect HIV status of the children included, as the study was implemented within an existing larger parent PCV13 effectiveness study. The cohort of CHWs that were originally selected to participate in the wider study were not trained or qualified to provide HIV care or use RDTs for malaria testing, something which is common in community settings. Prior studies have demonstrated that being HIV-infected or -exposed increases the odds of treatment failure in pneumonia (although these studies referred to severe pneumonia) [25, 26]. Malawi is considered a high HIV setting, with 8.9% of adults living in rural areas HIV-infected [18]. Currently, co-trimoxazole is given as a prophylaxis to HIV-infected infants; therefore some of the infants included in our cohort may have been taking co-trimoxazole already when diagnosed with fast-breathing pneumonia. Including HIV status in a prognostic model may improve its performance; however as the aim was to develop a tool that CHWs could feasibly implement in the field, including HIV status was not appropriate or practical for this cohort of government-supported CHWs. Ongoing work in Malawi suggests that closely supervised CHWs supported by the private sector can provide high quality HIV services, including pediatric HIV testing, therefore inclusion of HIV in prediction models may be more realistic in the near future for children cared for by select groups of CHWs with enhanced support [27]. The generalizability and acceptability of a model designed for use by rural CHWs that requires HIV status remains an important question.

We found concurrent clinical malaria diagnosis to be a predictor of antibiotic treatment failure. A study from Mozambique found that malaria diagnosis was the strongest risk factor associated with having an acute respiratory infection (ARI) diagnosis in infants [28]. Prior studies, including in Malawi, have demonstrated considerable overlap between malaria and pneumonia diagnosis based on clinical definitions, such as those used by the CHWs [29, 30]. A study from Zambia demonstrated a decrease in concurrent malaria and fast-breathing pneumonia diagnoses from 87% to 28% with the introduction of malaria RDTs [31], emphasizing this issue. Clinical diagnosis at the community level, especially in high HIV and malaria endemic areas, poses a challenge to pneumonia case-management [32]. It is probable that our cohort of ‘fast-breathing pneumonia’ patients included children with malaria who met the case definition for fast-breathing pneumonia and were treated as such, explaining why a concurrent clinical malaria diagnosis was associated with a poorer outcome. Future related work in malaria-endemic areas should consider the use RDTs at the community-level for malaria diagnosis, even if this is not routine amongst CHWs.

We excluded infants who were non-adherent from the analysis, based on information given by the care-giver and where possible, visibly counting remaining tablets. However, in this setting maternal education and literacy are low (35% completely illiterate [18]) and it is possible that we retained non-adherent children through poor caregiver understanding and reporting, and blood or urine testing for drug levels was not feasible. As we conducted this study in a quasi-programmatic setting, an issue worth considering is counterfeit drugs, with some pilot evidence that this is an issue in Malawi [33]. Adjusting for antibiotic quality and socio-economic factors may improve the performance of a prognostic model, but again would be impractical to include in a community-level referral tool.

This study had many strengths, in that it was a prospective community-based cohort implemented in a quasi-programmatic setting, generating evidence not only on outcomes but also the practicality of developing a reliable pragmatic prognostic without laboratory support. It also included the use of pulse oximetry at the CHW level to support diagnosis and referral decision-making. However, our key limitations were the lack of HIV and malaria testing and relatively high loss to follow-up, despite dedicated VDCs. Cases were recruited into the cohort during village clinics, where the VDCs would arrange to visit the patient’s house for follow up. Despite VDCs being recruited from the local area, there were issues in locating households, and families being unavailable. The challenges with the active follow-up, included: lack of consistent address information; people using multiple names; follow ups taking place over the rainy season making travel challenging, and during planting and harvesting time when care-givers were busy farming. Table 1 demonstrates there were some statistically significant differences at baseline between those with and without completed follow up, namely PCV13 receipt and moderate malnutrition. Those children lost to follow up were marginally better nourished, but had lower PCV13 coverage so it is unclear if having included these children would have changed the model. However, the model retained a sufficient number of treatment failures in relation to degrees of freedom; therefore, we do not think the high loss to follow-up detracts from the findings but rather highlights the difficulty of working in rural communities in a programmatic setting.

We have learnt several lessons in attempting to prospectively evaluate risks of treatment failure in clinically diagnosed pneumonia at the community level. Firstly, the reliability of CHW clinical diagnosis and treatment/referral decisions is a challenge and, despite active mentorship, misclassification and therefore incorrect treatment was common. Malawi guidelines do allow for CHWs to provide HIV testing services and recommend the use of RDTs but this is only realistically implemented with private support. Nevertheless, to address these challenges future studies should consider recruiting cohorts from higher levels of the healthcare system with access to malaria testing, HIV status and more qualified healthcare staff, although this may limit generalizability to the community setting. Secondly, our choice of VDCs was based on their local knowledge of the communities and not on clinical experience; in spite of this, we still had high loss to follow-up. Future studies could consider incentivizing care-givers to return to the healthcare facility for follow-up assessments as active community-based follow-up poses complex challenges, despite significant efforts. As co-trimoxazole is no longer the WHO recommendation for outpatient pneumonia treatment and newly revised guidelines allow for home treatment of children with lower chest indrawing, further research on the performance of a prognostic algorithm is needed.

While we have hypothesized several reasons for our findings, there is a lack of evidence around rates, causes and predictors of treatment failure in fast-breathing pneumonia at the community level in sub-Saharan Africa, with which to compare this study [17]. The differing etiologies, epidemiology and distribution of socio-economic risk factors between a rural sub-Saharan setting and South Asia [34], make it plausible that drivers of treatment failure (and therefore the predictors) would also be different. The potential for enhancing referral algorithms at the community level is exciting, but further work is needed to generate models with greater sensitivity.

## Supporting Information

S1 Text Analysis Plan. (DOCX)
