# Correction: Can We Predict Oral Antibiotic Treatment Failure in Children with Fast-Breathing Pneumonia Managed at the Community Level? A Prospective Cohort Study in Malawi

**DOI:** 10.1371/journal.pone.0139295

**Published:** 2015-09-24

**Authors:** Carina King, Eric D. McCollum, Limangeni Mankhambo, Tim Colbourn, James Beard, Debbie C. Hay Burgess, Anthony Costello, Rasa Izadnegahdar, Norman Lufesi, Gibson Masache, Charles Mwansambo, Bejoy Nambiar, Eric Johnson, Robert Platt, David Mukanga

The eighth author’s name is spelled incorrectly. The correct name is: Rasa Izadnegahdar.
