# Supplementary material for: Can We Predict Oral Antibiotic Treatment Failure in Children with Fast-Breathing Pneumonia Managed at the Community Level? A Prospective Cohort Study in Malawi
Source: PLoS One. 2015 Aug 27;10(8):e0136839. doi: 10.1371/journal.pone.0136839 (PMC4551481; doi:10.1371/journal.pone.0136839)
Supplement: S1 Text — (DOCX) [file pone.0136839.s001.docx]

**Analysis plan for fast-breathing pneumonia treatment failure prediction model**

Draft: October 3, 2013

This analysis plan outlines the steps required to develop an internally valid prediction model that will also meet journals’ expectations for reporting:^1^

(1) Identification of candidate predictor characteristics;

(2) Development of the prediction model using logistic regression;

(3) Assignment of the weights or risk score points for each predictor;

(4) Assessment of the prediction effectiveness;

(5) Internal validation.

We added an additional step that may be relevant to achieve the target product profile for the prediction model or algorithm: simplifying the model. This analysis plan includes a rationale for the decisions along with recent reviews on the essential methods. The R statistical code will be integrated into each step with further annotation.

Identification of candidate predictor characteristics

Candidate predictors will be identified from published literature and expert opinion. When the data have been collected in the prospective cohort study in Malawi, we’ll inspect their prevalence or distribution and percentage of missing values. We should consider excluding candidates with a low prevalence (e.g., <5%) or insufficient variability because those candidates will not contribute meaningfully to the prediction and we will lack confidence (precision) in the odds ratio that determines the risk score points.

Candidate predictors with a high (≥10%) percentage of missing values may not be suitable for a pragmatic prediction tool that will be used in the community because health workers may not be able to calculate the score and predict the risk of treatment failure:

“Another consideration with missing data is whether a variable that issued to develop the score and is frequently missing in the study may also be unavailable in the population to which the score will later be applied. If so, it is sensible to omit it from consideration in the prediction model.”^1^

For candidate predictors with limited (<10%) missing values, we may wish to impute values for the development cohort based on the observed variables collected via the case report form, including the treatment failure outcome.^2^ Statisticians have argued for multiple imputation for developing prediction models because imputation preserves the effective study size, which allows for the evaluation of more candidate predictors, and imputation also allows for more precise odds ratios (risk score points).^3,4^ Alternatively, we could develop the prediction model using a completed cases strategy (i.e., dropping patients with missing values for any candidate predictors).

After dropping candidate predictors with low prevalence/insufficient variability or a high percentage of missing values, we’ll undertake a strategy for missing data: multiple imputation or completed cases. Among the remaining candidate predictors, we’ll need to confirm that we have a sufficient study size. We must observe at least 10 treatment failures per degree of freedom for each candidate predictor evaluated if we wish the predictive algorithm or risk score to validate in subsequent populations.^5^ So 100 treatment failures would allow us to evaluate candidates from age through persistent vomiting or diarrhea (i.e., 10 degrees of freedom). The rule of 10 failures per degree of freedom protects against over-fitting the development data. We may need to drop one or more of the lower-priority predictors, or highly correlated predictors. Alternatively, we could continue collecting data to observe a sufficient number of treatment failures. Patients lost to follow-up for treatment failure may also reduce the study size.

Developing the prediction model

We will fit the fullest logistic regression model the available data allow using all of the candidate predictors noted above instead of applying variable selection strategies.^1,6,7^ For example, we will not simplify the model by omitting predictor characteristics based on their P-values.^6,7^ We are in the fortunate position that the full model is based on prior prediction estimates from earlier studies. However, because we expect a modest number of treatment failures, we cannot afford the degrees of freedom to explore non-linear relations (apart from the indicator variables for respiratory rate) or interactions. If we collected more data—say, 150 treatment failures—then we would explore non-linear relations for age with a restricted cubic spline on 3 knots (at the 10^th^, 50^th^ and 90^th^ percentiles), which would allow us to fit a flexible, smooth, non-linear relation between age and the risk of treatment failure. A restricted cubic spline on 3 knots would require an additional two degrees of freedom or 20 failures. Additional degrees of freedom could be spent exploring age by respiratory rate interactions. The required number of degrees of freedom would depend on whether age exhibited a linear or non-linear effect.

Assigning the relative weight (risk score points) for predictors

The logistic regression model’s coefficients for the predictors will serve as the weights for their relative importance in predicting the absolute, 5-day risk of treatment failure. The regression equation can be solved to obtain the exact failure probability for any patient in the cohort. For the sake of transparency and practicality, the coefficients are typically converted into an integer-based points system that approximates the relative weight of each predictor.^8^ Harrell’s R code will generate that risk scoring assignment automatically. For example, a patient with normal breathing (OR=1.0 would be scored with zero risk score points. Patients with fast breathing (e.g., OR=1.3), versus normal breathing, may be scored with one risk score point—a slightly worse prognosis. Patients with *very* fast breathing (e.g., OR=2.0) may be scored with three risk score points. When the prediction tool is used in practice, a child can be scored according to all of his or her characteristics; a greater number of risk score points means a higher absolute risk of treatment failure. Harrell’s R code will produce the tables and nomograms that reveal the link between the number of risk score points and the risk or probability of treatment failure.

Assessing prediction effectiveness

The logistic regression prediction model will provide a predicted risk (probability) of the 5-day treatment failure for each patient in the cohort. Those probabilities will be plotted using a risk predictiveness curve with probability on the Y-axis and the percentile on the X-axis.^9^ For each quartile of predicted risk, we will plot the observed risk of treatment failure to see how closely the observed agrees with the predicted—the calibration of the prediction model.^1^ Although many investigators prefer deciles, we anticipate too few treatment failures for that degree of stratification. We will also calculate the Hosmer-Lemeshow goodness of fit test for calibration, although the test will not be adequately powered to reject unsuccessful calibration (e.g., if we missed important interactions).^1,10^ We will also calculate the calibration slope for shrinkage of the summary performance statistics (discussed below in the context of the area under the receiver operating characteristic curve in the “internal validation” section).

The logistic regression prediction model will also provide the area under the receiver operating characteristic curve (AUROC), a summary measure of the discrimination of the prediction model.^1,10^ The risk predictiveness curve will also allow us to compare the observed risk of treatment failure in the highest quartile versus the observed risk in the lowest quartile (e.g., a four-fold separation of high- and low-risk patients). The Pneumonia Team may also wish to define a threshold for high-risk based on a clinically meaningful absolute risk of failure (e.g., ≥20% 5-day risk).

We will summarize the effectiveness of the prediction model by calculating Nagelkerke’s R^2^, a measure of the explained variation in absolute risk.^10^

Internal validation

To assess the extent of over-fitting the prediction model and the resulting optimism of its performance, we will calculate summary statistical measures for the internal validation.^1^ Cross-validation with bootstrapping generates more statistically efficient measures than the older “hold-out” sample strategy.^1^ The AUROC and its 95% confidence interval will be corrected with bootstrap-resampling to account for any over-fitting and optimism in the predictions so that we have a more credible estimate of discrimination in future patients.^1,12^

Simplifying the prediction model

The target product profile for the treatment failure model seeks a prediction based on as few as three patient characteristics. While practical for use in the community, that level of simplicity may be infeasible statistically. Harrell developed a statistical approach for simplifying a full model into a simpler set of predictors based on each predictor’s contribution to the variance explained or R^2^.^13^ The Pneumonia Team can assess the decline in prediction effectiveness for each predictor omitted to determine the optimal simplification.

References

1. Moons KGM, Pengne AP, Woodward M, Royston P, Vergouwe Y, Altman DG, Grobbee D. Risk prediction models: 1. Development, internal validation, and assessing the incremental value of a new (bio)marker. Heart 2012;98:683-690.

2. Moons KG, Donders RA, Stijnen T, et al. Using the outcome for imputation of missing predictor variables was preferred. J Clin Epidemiol 2006;59:1092-1101.

3. Vergouwe Y, Royston P, Moons KG, Altman DG. Development and validation of a prediction model with missing predictor data: a practical approach. J Clin Epidemiol 2010;63:205-14.

4. Marshall A, Altman DG, Royston P, Holder RL. Comparison of techniques for handling missing covariate data within prognostic modeling studies: a simulation study. BMC Med Res Methodol 2010;10:doi:10.1186/1471-2288-10-7.

5. Steyerberg EW, Eijkemans MJC, Harrell FE, Habbema JDF. Prognostic modeling with logistic regression analysis: In search of a sensible strategy in small data sets. Med Decis Making 2001;21:45-56.

6. Steyerberg EW. Selection of main effects (Chapter 11). In: *Clinical Prediction Models: A Practical Approach to Development, Validation, and Updating*. Rotterdam: Springer, 2009.

7. Harrell FE. Multivariable modeling strategies (Chapter 4). In: *Regression Modeling Strategies*. New York: Springer, 2001.

8. Sullivan LM, Massaro JM, D’Agostino RB, Sr. Presentation of multivariate data for clinical use: the Framingham Study risk score functions. Statist Med 2004;23:1631-60.

9. Pepe MS, Feng Z, Huang Y, et al. Integrating the predictiveness of a marker with its performance as a classifier. Am J Epidemiol 2008;167:362-68.

10. Steyerberg EW, Vickers AJ, Cook NR, et al. Assessing the performance of prediction models: a framework for traditional and novel measures. 2010;21:128-38.

11. Hosmer DW, Hosmer T, Cessie LE, Lemeshow S. A comparison of goodness-of-fit tests for the logistic regression model. Stat Med 1997;16:965-80.

12. Steyerberg EW. Overfitting and optimism in prediction models (Chapter 5). In: *Clinical Prediction Models: A Practical Approach to Development, Validation, and Updating*. Rotterdam: Springer, 2009.

13. Harrell FE, Jr, Margolis PA, Gove S, et al. Development of a clinical prediction model for an ordinal outcome: The World Health Organization Multicentre Study of clinical signs and etiological agents of pneumonia, sepsis and meningitis in young infants. Stat Med 1998;17:909-44.
